# 

**Published:** 1908-12

**Authors:** 


### BOOKS RECEIVED.

Diseases and Surgery of the Genitourinary System. By Francis S. Watson, M.D., Senior Visiting Surgeon to the Boston City Hospital, Lecturer on Genitourinary Surgery in the Harvard Medical School, Boston, and John H. Cunningham, Jr., M.D., Assistant Visiting Surgeon to the Boston City Hospital. In two octavo volumes containing 1,101 pages, with 454 engravings and 47 full-page colored plates, mostly from original drawings. Lea & Febiger, Publishers, Philadelphia and New York, 1908. (Price for the complete work: Extra cloth, $12.00, net; half persian morocco, gilt tops, le luxe, $17.00, net.)

International Clinics. A Quarterly of Illustrated Clinical Lectures and especially prepared articles on Treatment, Medicine, Surgery, Neurology, Pediatrics, Obstetrics, Gynecology, Orthopedics, Pathology, Dermatology, Ophthalmology, Otology, Rhinology, Laryngology, Hygiene and other topics of interest to students and practitioners. Edited by W. T. Longcope, M.D., Volume II, eighteenth series. Philadelphia and London: J. B. Lippincott Co. 1908. (Cloth, $2.00.)

Applied Surgical Anatomy, Regionally Presented. By George Woolsey, A.B., M.D., Professor of Anatomy and Clinical Surgery in Cornell University Medical College, New York. Second edition. Octavo volume of 601 pages, with 200 illustrations in black and colors. Lea & Febiger, Philadelphia and New York, 1908. (Cloth, $4.50, net.)

Surgery: Its Principles and Practice. In five volumes. By 66 eminent surgeons. Edited by W. W. Keen, M.D., LL.D., Hon. F.R.C.S., Eng. and Edin., Emeritus Professor of the Principles of Surgery and of Clinical Surgery, Jefferson Medical College, Phila. Volume IV. Octavo of 1194 pages, with 560 text-illustrations and 22 colored plates. Philadelphia and London: W. B. Saunders Company, 1908. (Per volume: cloth, $7.00 net; half morocco, $8.00 net.)

Medical Directory of New York, New Jersey and Connecticut. Published by the Medical Society of the State of New York. Vol. X. 1908.

Transactions of the sixth annual conference of state and territorial health officers with the United States public health and marine hospital service, held at Washington, D. C., April 27, 1908.

Anatomy. By Dr. Carl Nicoladoni, Graz. Illustrated. Berlin: Urban & Schwarkenberg.

Therapeutics of the Circulation. Eight lectures delivered in the spring of 1905, at the University of London. By Lauder Brunton, Consulting Physican to St. Bartholomew's Hospital. Small 8vo. pp, 280. Illustrated. Philadelphia: P. Blakiston's Son & Co. ($1.50.)

Surgical Memoirs and Other Essays. By James G. Mumford, M.D., Instructor in Surgery, Harvard Medical School. 12 mo, pp. 358. Illustrated. New York: Moffat, Yard & Co. (Cloth, $2.50.)

Russell Sage Foundation. Medical Inspection of Schools. By Luther Halsey Gulick, M.D., Director of Physical Training, New York Public Schools, and Leonard P. Ayres, General Superintendent of Schools of Porto Rico, 1906, 1908. New York Charities Publication Committee. (Cloth, $1.00.)

Transactions of the Section on Obstetrics and Diseases of Women of the American Medical Association at the 59th annual session held at Chicago, June 2, 5, 1908.

Principles and Practice of Physical Diagnosis. By John C. DaCosta, Jr., Assistant in Clinical Medicine in Jefferson Medical College, Philadelphia. Octavo, pp. 548. Illustrated. Philadelphia and London: W. B. Saunders Co. (Cloth, $3.50.)

General Pathology. By Dr. Ernst Ziegler, Breisgau. Eleventh revised German edition. Edited by Aldred Scott Warthin, M.D., Ann Arbor. Octavo, pp. 801. Illustrated. New York: William Wood & Co. (Cloth, $5.50.)

The Surgery of the Ear. By Samuel J. Kopetzky, M.D., Attending Otologist, New York City Children's Hospitals and Schools. Octavo, pp. 385. Illustrated. New York: Rebman Co. (Cloth, $4.00.)

A Handbook of Suggestive Therapeutics, Applied Hypnotism, Psychic Science. By Henry S. Munro, M.D., Americus, Ga. 12 mo, pp. 360. Second editon. St. Louis: C. V. Mosby Co. (Cloth, $3.00.)

Practical Points in Anesthesia. By Frederick-Emil Neef, New York. Small 12 mo, pp. 51. New York: Surgery Publishing Co.

Arteriosclerosis. By Louis M. Warfield, M.D., Instructor in Medicine in Washington University Medical Department. 12 mo, pp. 165. Illustrated. St. Louis: C. V. Mosby Co. (Cloth, $2.00.)

Gonorrhea in Women. By Palmer Findley, M.D., Professor of Gynecology in the College of Medicine of the University of Nebraska, Omaha. pp. 112. St. Louis: C. V. Mosby Co. (Cloth, $2.00.)

Proceedings of the American Medico-Psychological Association at the sixty-third annual meeting held at Washington, D. C., May 7, 10, 1907. C. W. Pilgrim, M.D., Secretary.

